# Supplementary material for: Transient circular dichroism and exciton spin dynamics in all-inorganic halide perovskites
Source: Nat Commun. 2020 Nov 9;11:5665. doi: 10.1038/s41467-020-19471-9 (PMC7653957; doi:10.1038/s41467-020-19471-9)
Supplement: Supplementary file 1 — Supplementary Information [file 41467_2020_19471_MOESM1_ESM.pdf]

## Supplementary Information

# Transient circular dichroism and exciton spin dynamics in all-inorganic halide perovskites

Weijie Zhao[1#], Rui Su[1#], Yuqing Huang[1], Jinqi Wu[1], Chee Fai Fong[1], Jiangang Feng[1], Qihua Xiong[1,2*]

[1]Division of Physics and Applied Physics, School of Physical and Mathematical Sciences, Nanyang Technological University, Singapore 637371, Singapore.

[2]State Key Laboratory of Low-Dimensional Quantum Physics and Department of Physics, Tsinghua University, Beijing 100084, China.

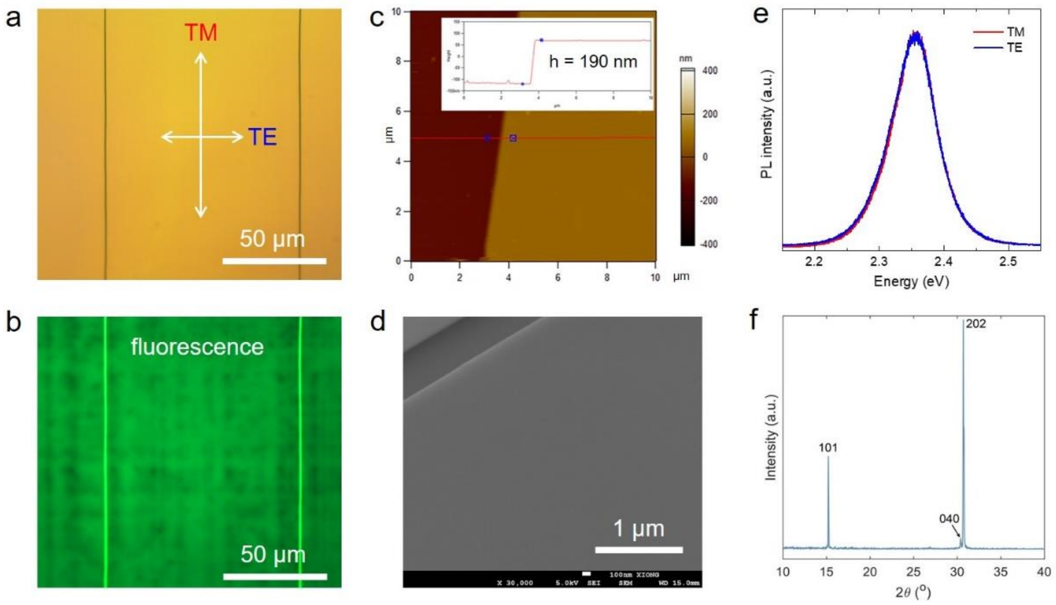


**Supplementary Figure 1. Sample characterizations. a** Optical image of single-crystal $CsPbBr_3$ thin films. The black lines are cracks or grain boundaries owing to the strain relaxation of $CsPbBr_3$ films on Mica substrates during the epitaxial growth [1]. **b** The fluorescence image for the sample shown in **a**. The homogeneous fluorescence intensity between the cracks or grain boundaries indicates the high-quality of the grown thin films. The dark dots or lines in the background are from the Mica substrate. **c** The AFM image reveals a thickness of ~190 nm and a surface roughness of ~1 nm for the sample used to obtain the TA spectra in the main text. The spin-contrasting optical nonlinearities of $CsPbBr_3$ did not show notable dependence on sample thickness in a wide range from ~100 to 300nm. **d** The high-magnification SEM image shows the flat surface and sharp edges of $CsPbBr_3$ thin films. **e** The PL spectra of the TE and TM modes [2] indicated by the white arrows in **a**. **f** The high-resolution XRD pattern of $CsPbBr_3$ thin film exhibits typical diffraction peaks of (*101*), (*040*), (*202*) planes. The splitting of (*202*) diffraction peak suggests the orthorhombic structure. To the best of our knowledge, the XRD pattern of our perovskite films displays the narrowest FWHM of 0.07° among the literatures [1,3,4], which justifies the superior crystallinity of our perovskite films.

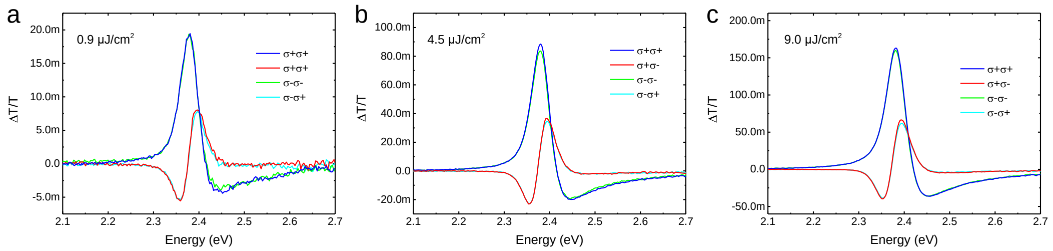


**Supplementary Figure 2. TA spectra of $CsPbBr_3$ obtained with different pump-probe configurations at 0.3 ps at room temperature. a-c** The pump fluence are ~0.9, 4.5 and 9.0 μJ/cm$^2$ , respectively. The photon energy of pump beam is ~2.4 eV, which is in resonant with the exciton states. The differences between the σ-σ- (σ-σ+) and σ+σ+ (σ+σ-) TA spectra are less than 5% at high pump fluence and varies randomly in multiple measurements, which is attributed to experimental errors caused by the manually rotated quarter- waveplate with a accuracy of $\pm 0.5^\circ$ .

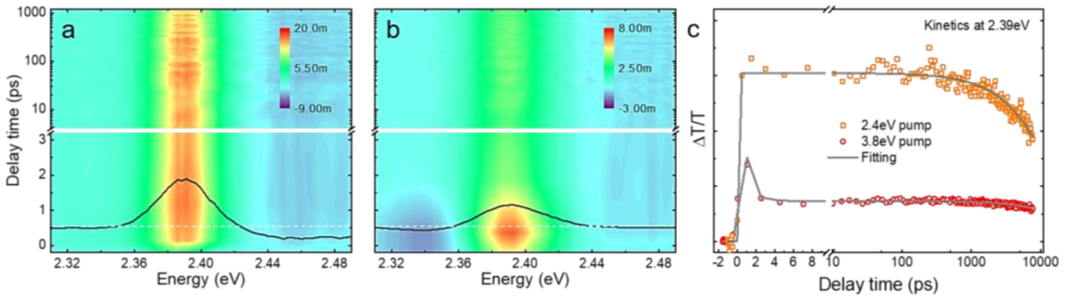


**Supplementary Figure 3. TA spectra with linearly polarized pump and probe beams. a, b** Time evolution of TA spectra of $CsPbBr_3$ with resonant (~2.4 eV) and non-resonant (~3.8 eV) excitations, respectively, at room temperature. The pump and probe pulses are cross-linearly polarized. The pump fluence is ~0.9 μJ/cm$^2$ . The black lines are TA spectra at ~0.5 ps. **c** TA kinetics at ~2.39 eV fitted with single- or bi-exponential decays. Because of the direct injection of non-polarized excitons into the system for the resonant excitation, TA spectra are the same as those shown in Fig. 1b and 1c at delay times longer than 50 ps in the main text. While for the high above bandgap excitation, a significant PIA feature appears at the probe energy range of 2.36-2.28 eV and is linked to the hot carrier caused energy renormalization [5,6]. The short lifetime (~0.6 ps) of this PIA suggests strong electron-phonon couplings in $CsPbBr_3$ at room temperature [7].

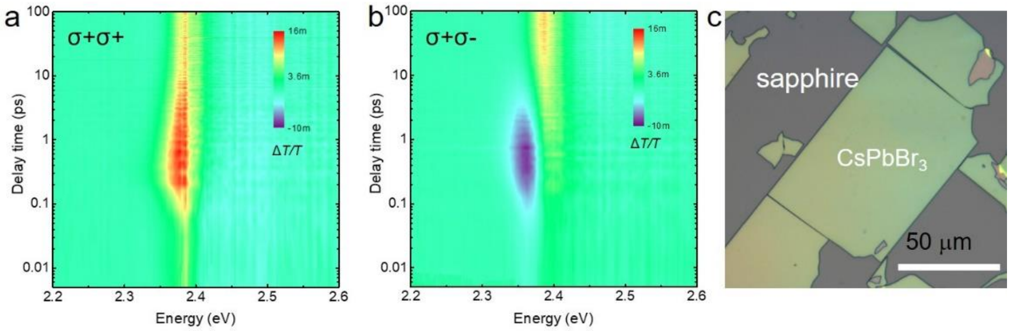


**Supplementary Figure 4. Time evolution of TA spectra of $CsPbBr_3$ on sapphire substrate.** **a**,**b** The TA spectra are obtained with the σ+σ+ and σ+σ- pump-probe configuration, respectively, at room temperature. The photon energy of pump beam is ~2.4 eV. The pump fluence is ~0.8 μJ/cm$^2$ . **c** An exemplary optical image of $CsPbBr_3$ thin films transferred onto double-polished sapphire wafer.

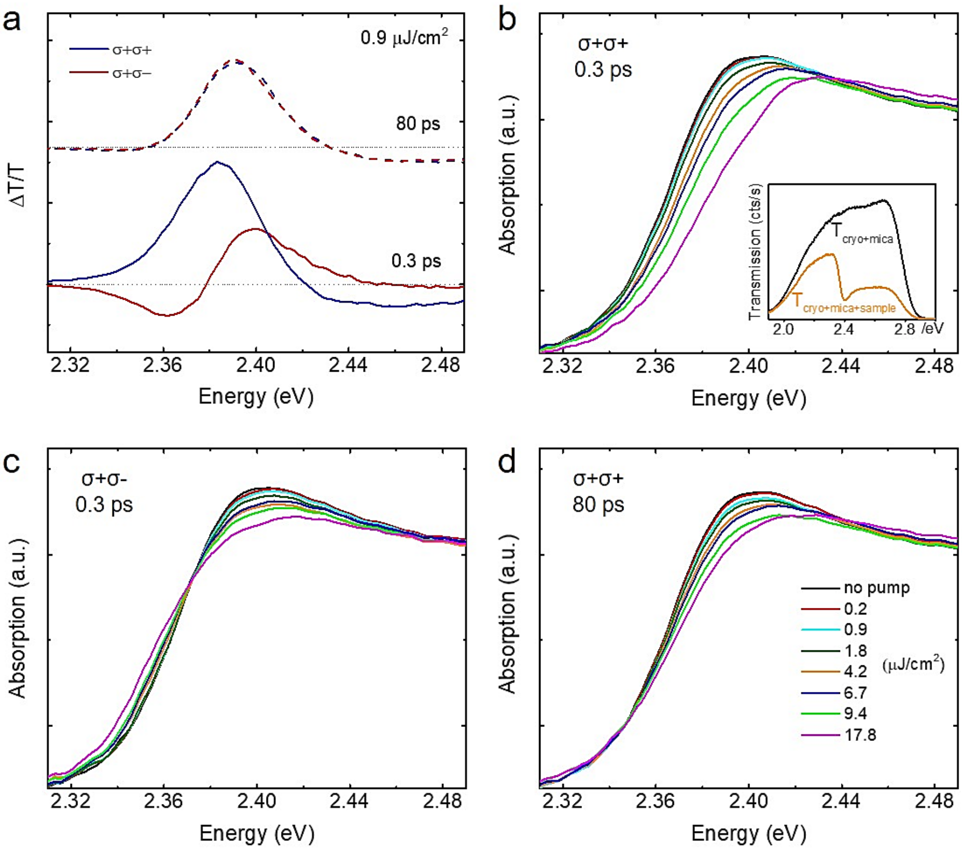


**Supplementary Figure 5. Pump fluence dependent TA and absorption spectra. a** TA spectra at different delay times extracted from Fig. 1b and 1c in the main text. At 80 ps, the σ+σ+ and σ+σ- spectra are identical. **b-d** Absorption spectra at different pump-probe

configuration or delay times as a function of pump fluence at room temperature. We did not observe any notable difference between the σ+ and σ- absorption spectra without pump. The inset to **b** shows the transmission spectra of the probe beam with and without perovskite sample. The absorption spectra are calculated as the differential transmission at different delay times. The contributions from pump-induced changes of reflectance are neglected since they do not affect general results of optical nonlinearities [8]. In **a**, we noticed that there are tiny PIA features in the σ+σ- TA spectra at 0.3 ps (the solid red line) at the high-energy side and also in the TA spectra at 80 ps at the low-energy side, which are possibly from the exciton broadening effect as a result of spin-sensitive exciton scatterings [9,10]. $\Delta E$ can be ~23.0 meV at a pump fluence of 17.8 μJ/cm$^2$. In **d**, when exciton spins fully relax, the blueshift of exciton resonance are much smaller than those shown in **b**.

At high pump fluence, the electronic phase transition between the exciton and free-carrier states (i.e., the excitonic Mott transition) would happen. The Mott density ($n_{\mathrm{M}}$) based on the Mott criteria [$(n_{\mathrm{M}})^{1/3}\cdot a_{\mathrm{B}} \approx 0.2$, where $a_B$ is the exciton Bohr radius] [11]. The $n_{\mathrm{M}}$ is ~1.8 x $10^{17}$ cm$^{-3}$ calculated with an exciton Bohr radius of 3.5 nm in bulk $CsPbBr_3$ [12]. However, the Mott criteria is derived based on the screening effect from free carriers [11]. Here we directly inject excitons in the system by using resonant excitations. The screening effect from these charge neutral quasiparticles (excitons) is about one order of magnitude weaker than that of free carriers [13]. Therefore, the actual Mott density could be ~1.8x$10^{18}$ in $CsPbBr_3$, which is corresponding to a pump fluence of ~31.1 μJ/cm$^2$ if assuming that the absorption of the pump beam is in the linear regime. Therefore we can safely claim that the spin splitting are primarily from the many-body interaction of spin-polarized excitons with pump fluence below ~10 μJ/cm$^2$. Indeed, the energy splitting between +1 and -1 exciton states, as well as the bleaching of the exciton absorption, show a linear increase with the increase of pump fluence (< 10 μJ/cm$^2$). In addition, the above analysis is undoubtedly supported by a recent paper [5], which studied the room-temperature TA spectra of methylammonium lead iodide ($MAPbI_3$) polycrystalline films experimentally and theoretically. Despite of a small exciton binding energy

of ~9 meV in $MAPbI_3$, the saturation effect and the excitonic Mott transition are observed to happen at a large excitation density of ~$1x10^{18}$ $cm^{-3}$ [5]. Therefore, the excitonic Mott transition would occur at an excitation density larger than ~$1x10^{18}$ $cm^{-3}$ in $CsPbBr_3$ owing to the large exciton binding energy of ~40 meV [2].

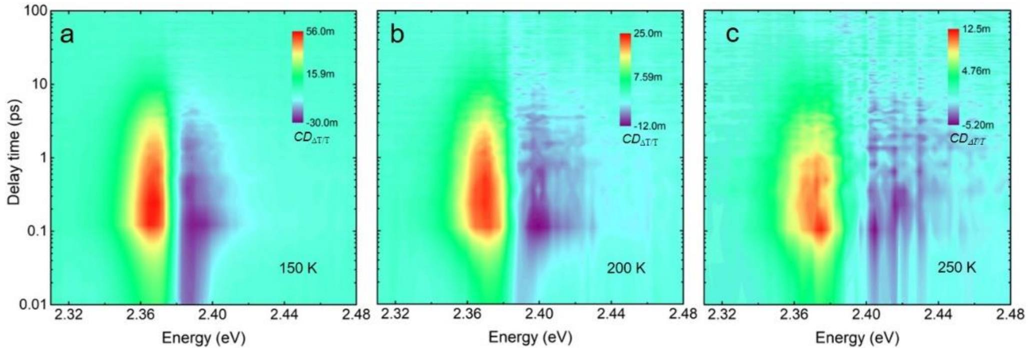


**Supplementary Figure 6. Temperature dependent TA spectra. a-c** The circular dichroism signal ($CD_{\Delta T/T}$) at 150, 200 and 250 K, respectively, under resonant excitations, showing similar exciton spin dynamics. The pump fluence was kept at ~0.2 μJ/$cm^2$ .

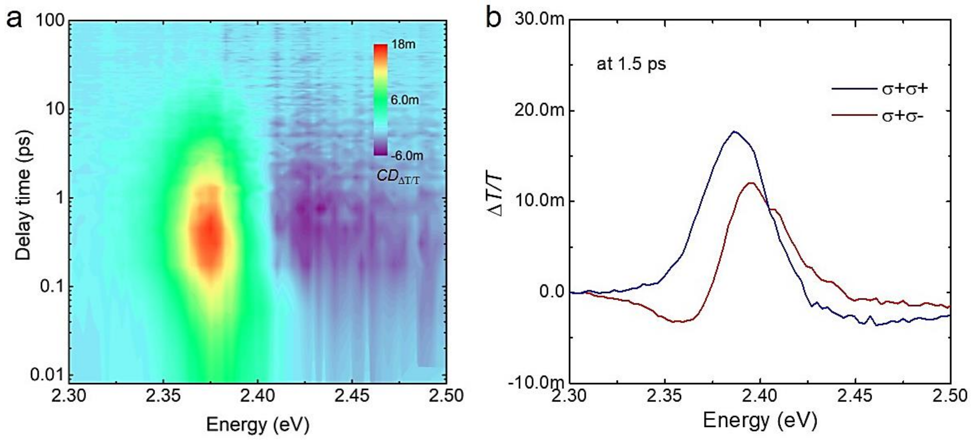


**Supplementary Figure 7. TA spectra under a non-resonant excitation. a** The circular dichroism signal ($CD_{\Delta T/T}$) with a non-resonant excitation at ~2.6 eV at room temperature. The pump fluence is ~0.9 μJ/$cm^2$ . **b** The σ+σ+ and σ+σ- TA spectra at ~1.5 ps when the hot carriers fully relax and the exciton population builds up, corresponding to a circular polarization degree of ~30%.

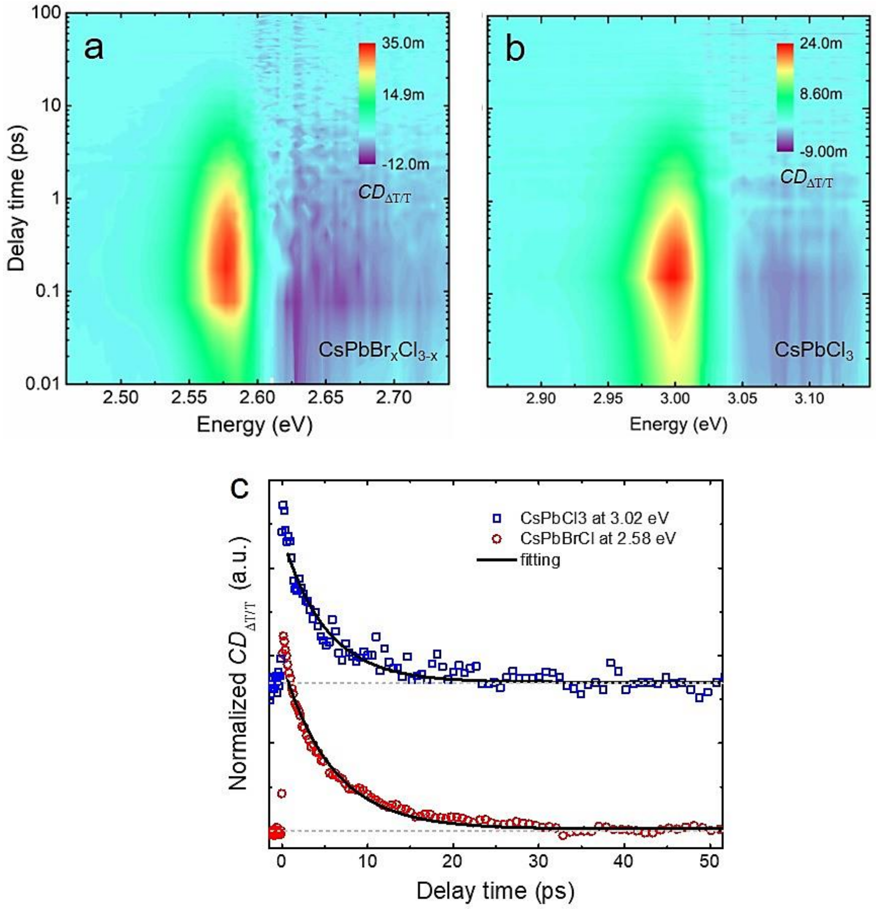


**Supplementary Figure 8. TA spectra of single-crystal $CsPbBr_xCl_{3-x}$ and $CsPbCl_3$ thin films.**

**a,b** The circular dichroism signal ( $CD_{\Delta T/T}$ ) of single-crystal $CsPbBr_xCl_{3-x}$ and $CsPbCl_3$, respectively, under resonant excitation at room temperature. The pump fluence is ~0.9 μJ/cm$^2$ .

**c** The kinetics of $CD_{\Delta T/T}$ extracted from (a) and (b) are fitted with single exponential decay from ~0.6 ps to avoid the fast decay component as discussed in the main text. The horizontal dashed lines are the base lines with $\Delta T / T = 0$ . The obtained exciton spin decay time ( $\tau_{\mathrm{Wex}}$ ) for $CsPbBr_xCl_{3-x}$ and $CsPbCl_3$ are ~5.9 and 4.9 ps, respectively. The $\tau_{\mathrm{Wex}}$ in $CsPbCl_3$ is slightly shorter than those in $CsPbBr_3$ (~6.2 ps, as shown in Fig. 3 in the main text) and $CsPbBr_xCl_{3-x}$ under the same pump fluence, which is probably due to its stronger excitonic effects [14]. As the exciton binding energy in $CsPbCl_3$ is ~70 meV [14], the *e*-*h* exchange interaction within the exciton is expected to be stronger than that in $CsPbBr_3$.

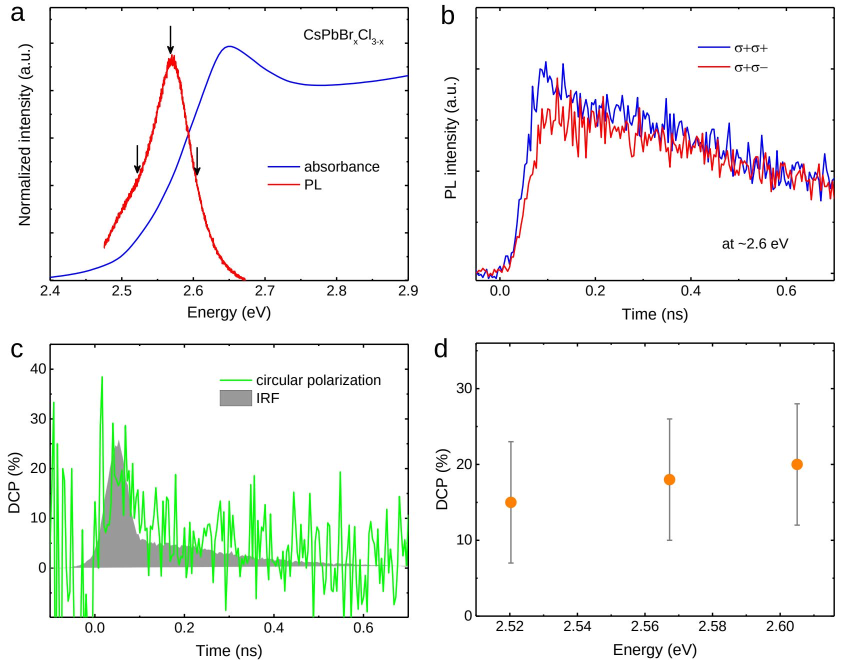


**Supplementary Figure 9. Time-resolved PL decays. a** Room-temperature PL (red curve) and absorbance (blue curve) spectra of the $CsPbBr_xCl_{3-x}$ thin film. The large emission Stokes (~80 meV) shift is not due to defects or impurities but from the lattice anharmonicity in carrier-lattice interactions which is intrinsic to metal halide perovskites [15]. The three black arrows indicate the emission energy which are selected to check the PL decay. **b** The PL decay curves with the σ+σ+ (blue curve) and σ+σ- (red curve) excitation-detection polarization configuration at ~2.6 eV. **c** The degree of circular polarization (DCP) calculated based on the PL decay curves shown in **b**. The instrument response function (IRF) measured at ~2.6 eV is shown as a comparison (shaded grey). **d** The DCP obtained at three emission energies indicated by the black arrows in **a**. The maximum DCP at ~16 ps is relatively low (~20 ± 8%) because the PL decay curve is measured under a non-resonant excitation, which is consistent with the observations by using the transient absorption spectra shown in Fig. 4b in the main text.

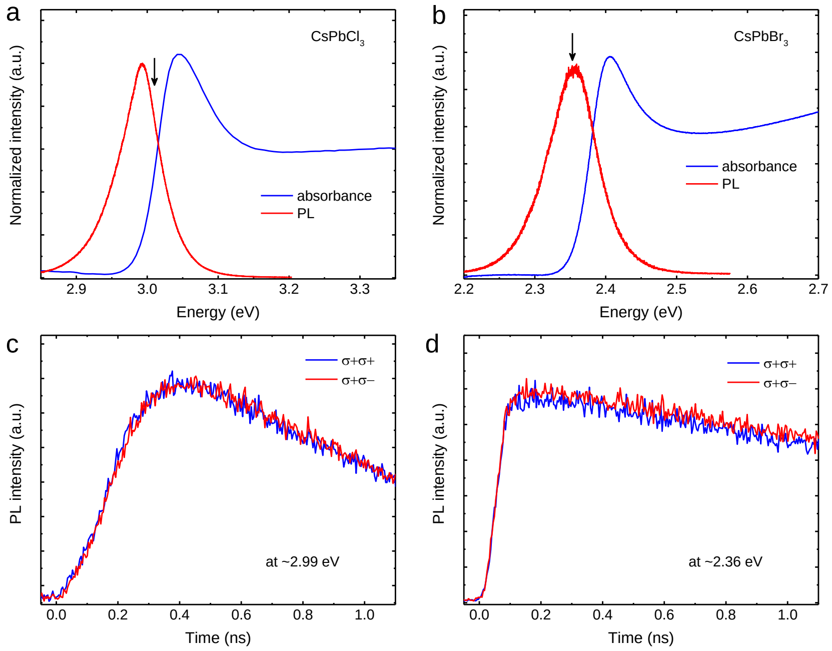


**Supplementary Figure 10. Time-resolved PL decays.** Room-temperature PL (red curve) and absorbance (blue curve) spectra of $CsPbCl_3$ **a** and $CsPbBr_3$ **b** thin films. The PL decay curves with the σ+σ+ (blue curve) and σ+σ- (red curve) excitation-detection polarization configuration at ~2.99 eV for the $CsPbCl_3$ **c** and at ~2.36 eV for $CsPbBr_3$ **d** thin films. In **c**, a very long rising time (~400 ps) is observed in the PL kinetics of $CsPbCl_3$ thin films. This indicates that the conversion process from the absorption to emission states are very slow. Therefore, excitons lost their spin orientation within this long conversion process. In **d**, the exciton spin dynamic in the PL decay curves is missing in $CsPbBr_3$ because the excitation energy is too far away from the exciton states and initially leads to a very low spin injection efficiency of excitons.

## Supplementary References:

[1] Wang, Y. et al. Nontrivial strength of van der Waals epitaxial interaction in soft perovskites. *Phys. Rev. Mater.* **2**, 076002 (2018).

[2] Su, R. et al. Observation of exciton polariton condensation in a perovskite lattice at room temperature. *Nat. Phys.* **16**, 301-306, (2020).

[3] Rakita Y. et al. Low-Temperature Solution-Grown $CsPbBr_3$ Single Crystals and Their Characterization. *Cryst. Growth Des.* **16**, 5717−5725 (2016)

[4] Yang Z. et al. Large and Ultrastable All-Inorganic $CsPbBr_3$ Monocrystalline Films: Low-Temperature Growth and Application for High-Performance Photodetectors. *Adv. Mater.* **30**, 1802110 (2018).

[5] Yang, Y. et al. Observation of a hot-phonon bottleneck in lead-iodide perovskites. *Nat. Photon.* **10**, 53-59 (2016).

[6] Richter, J. M. et al. Ultrafast carrier thermalization in lead iodide perovskite probed with two-dimensional electronic spectroscopy. *Nat. Commun.* **8**, 376 (2017).

[7] Evans, T. et al. Competition Between Hot-Electron Cooling and Large Polaron Screening in $CsPbBr_3$ Perovskite Single Crystals. *J. Phys. Chem. C* **122**, 13724 (2018).

[8] Price, M. B. et al. Hot-carrier cooling and photoinduced refractive index changes in organic–inorganic lead halide perovskites. *Nat. Commun.* **6**, 8420 (2015).

[9] Smith, R. P. et al. Extraction of Many-Body Configurations from Nonlinear Absorption in Semiconductor Quantum Wells. *Phys. Rev. Lett.* **104**, 247401 (2010).

[10] Le Jeune, P. et al. Spin-dependent exciton-exciton interactions in quantum wells. *Phys. Rev. B* **58**, 4853-4859 (1998).

[11] Mott N. F., Metal-insulator transition. *Rev. Mod. Phys.* **40**, 677 (1968).

[12] Isarov, M. et al. Rashba Effect in a Single Colloidal $CsPbBr_3$ Perovskite Nanocrystal Detected by Magneto-Optical Measurements. *Nano Lett.* **17**, 5020-5026 (2017).

[13] Schultheis, L. et al. Ultrafast Phase Relaxation of Excitons via Exciton-Exciton and Exciton-Electron Collisions. *Phys. Rev. Lett.* **57**, 1635-1638 (1986).

[14] Zhang, Q. et al. High-Quality Whispering-Gallery-Mode Lasing from Cesium Lead Halide Perovskite Nanoplatelets. *Adv. Funct. Mater.* **26**, 6238 (2016).

[15] Guo Y. et al., Dynamic emission Stokes shift and liquid-like dielectric solvation of band edge carriers in lead-halide perovskites. *Nat. Commun.* **10**, 1175 (2019).
